# Quality of science journalism in the age of Artificial Intelligence explored with a mixed methodology

**DOI:** 10.1371/journal.pone.0303367

**Published:** 2024-06-18

**Authors:** Anne M. Dijkstra, Anouk de Jong, Marco Boscolo

**Affiliations:** 1 Department of Communication Science, University of Twente, Enschede, the Netherlands; 2 Formicablu, Bologna, Italy; Department of Physics, University of Bologna, Bologna, Italy; University of Haifa, ISRAEL

## Abstract

Science journalists, traditionally, play a key role in delivering science information to a wider audience. However, changes in the media ecosystem and the science-media relationship are posing challenges to reliable news production. Additionally, recent developments such as ChatGPT and Artificial Intelligence (AI) more generally, may have further consequences for the work of (science) journalists. Through a mixed-methodology, the quality of news reporting was studied within the context of AI. A content analysis of media output about AI (news articles published within the time frame 1 September 2022–28 February 2023) explored the adherence to quality indicators, while interviews shed light on journalism practices regarding quality reporting on and with AI. Perspectives from understudied areas in four European countries (Belgium, Italy, Portugal, and Spain) were included and compared. The findings show that AI received continuous media attention in the four countries. Furthermore, despite four different media landscapes, the reporting in the news articles adhered to the same quality criteria such as applying rigour, including sources of information, accessibility, and relevance. Thematic analysis of the interview findings revealed that impact of AI and ChatGPT on the journalism profession is still in its infancy. Expected benefits of AI related to helping with repetitive tasks (e.g. translations), and positively influencing journalistic principles of accessibility, engagement, and impact, while concerns showed fear for lower adherence to principles of rigour, integrity and transparency of sources of information. More generally, the interviewees expressed concerns about the state of science journalism, including a lack of funding influencing the quality of reporting. Journalists who were employed as staff as well as those who worked as freelancers put efforts in ensuring quality output, for example, via editorial oversight, discussions, or memberships of associations. Further research into the science-media relationship is recommended.

## Introduction

Science journalists, traditionally, play a key role in delivering quality science information to a wider audience. Not only by disseminating information. Media also enable participation in discussions and public debates [1] and have an agenda setting influence [2]. According to Dunwoody [3] access to high-quality information about science and technology is vital since the way people encounter scientific content matters. A media ecosystem producing quality information is therefore crucial. It is expected that recent developments within the field of Artificial Intelligence (AI) will increasingly impact the existing media ecosystems [4]. This is particularly true since the arrival and the rising popularity of chatbots based on Large Language Models (LLM) such as ChatGPT, Bard and Midjourney [5].

The past years have already seen several changes influencing the field of science journalism, quality reporting and the science-media relationship in general [cf. 1–6]. For example, the media ecosystem has undergone major changes where barriers between traditional producers and users of information–citizens–are nowadays lower and mingled. Currently, everyone can produce media content and contribute to different narratives about science. Additionally, the rise of mis- and disinformation has been significant [7], putting pressure on the responsibility of science journalists and the profession of science journalism more broadly, while the profession increasingly has had to deal with financial challenges leading to additional time-constraints when producing science information [7–9].

The precise effects of these developments for the quality of science reporting vary per country and depend also on politics and media culture [10]. While De Jong & Dijkstra reported that most studies in their literature review on the science-media relationship only included Western perspectives from a few countries, mostly the UK or the US [11], see recently [12], Schäfer suggested not to limit studies to mainly Anglo-Saxon countries [4]. In fact, Southern-European perspectives have been understudied [cf. 4, 11], as is the topic of AI related to science reporting and science journalism [cf.5, 12], which is why our research focused on reporting on AI in Italy, Portugal, Spain and Belgium. Belgium is another understudied area which houses the headquarters of the EU commission in Brussels.

In sum, in two studies we explored the reporting about AI to better understand the media ecosystems regarding science reporting in the four countries. Our aims were to, first, analyse how–in the four countries—quality criteria for science journalism were reflected in news reporting about AI. Therefore, we analysed news output and, in particular, conducted a qualitative in-depth content analysis of a sample of those news articles about AI in Belgium, Italy, Portugal and Spain. Our second aim was to gather the perspectives of journalists on the quality of reporting and science journalism in the context of recent developments of AI generated content and past developments in the field. We interviewed science journalists from the four countries. The mixed-method approach provided us with more nuanced insights than a single study could have achieved [13]. Our research questions are as follows:

1. How is quality in news reporting reflected in the content of news articles about AI in Belgium, Italy, Portugal and Spain?
2. How do journalists in these countries, within the context of current developments of AI and the past developments in the field, deal with quality of science journalism in practice?
3. What can be learned from comparing the media ecosystems regarding the quality of science journalism in Belgium, Italy, Portugal and Spain?

### Changing media landscapes

Nowadays, news is increasingly spread via social media rather than via traditional media such as newspapers, television, or radio. This also applies for science news. Speaking about science journalism, Dunwoody [3] and Brown [14] have argued that traditional boundaries between sources, journalists and audiences have been fading, which changed the landscape of science journalism. For example, audiences expect continuous updates on news instead of, as it was traditionally, updates of once a day (newspapers), once an hour (television) or once every half hour (radio) at the most [1]. The urgency to make news available at any moment, makes it more difficult for science journalists to adhere to their traditional tasks of checking the quality of information and providing context. Also, social media enable others to produce science knowledge any time without contributions from intermediaries such as journalists. Together with lower incomes generated from advertisements, consequently, traditional business models have increasingly become less sustainable, and positions for science journalism have become less stable and less available [4].

Developments within the field of AI are expected to further contribute to changes in the media ecosystems. First coined in 1956, in current definitions AI refers to systems enabling problem-solving and decision-making or displaying intelligent autonomous behaviour [15] and can be software-based as well as embedded in hardware devices, as stated in the definition from the High Level Expert Group from the European Commission on AI [16]. With the launch of ChatGPT on 30 November 2022, the use of generative AI has started dominating discussions about societal implications in the public domain. ChatGPT, which is a Large Language Model (LLM), was trained to provide detailed responses to prompts in a conversational way [17]. Its applications are multiple. It reached a million users in less than a week and 100 million users in January 2023 [5]. Expectations are that ChatGPT and comparable tools that can generate and translate text, create images, or imitate voices, will impact not only society but also academia and the sciences, as well as have consequences for media and the science communication field more broadly, and for science journalists, and the quality of science journalism more specifically [5, 18, 19].

Expected benefits of generative AI tools, from a user perspective, according to Schäfer (p.3), are an ability to ‘provide dialogical science communication at scale’, which would make science available for everyone and thus has a potential for the practice of science communication [5]. Optimists, for now, see mainly such benefits, while pessimists point at challenges with, for example, accuracy of the information, wrong or fictitious references, the quality of the information, and predict a new infodemic which will be AI driven and can exponentially increase mis- or disinformation and knowledge fabrication or hallucinations [5, 15]. Furthermore, the developments have strengthened concerns about job security including those in science journalism [5, 20], thus, leading to considerable challenges for science journalists.

Such challenges and concerns were, for example, also discussed at the International Journalism Festival which was held in April 2023 in Perugia in Italy [19] and in recent news articles in various countries about the impact of generative AI for journalism [18, 20–22]. Benefits, such as the ability to produce large amounts of texts were identified, while risks were also brought up, including doubts about the reliability of the information, for example, by chatbots referring unjustly to reliable news sources and made-up information, therewith, also attacking the trustworthiness of traditional media outlets [18, 19, 22]. Another concern regards the influence on the economic models of journalism since the AI tools can produce information that normally traditional media would produce. It can further deteriorate the viability of news producers and the work of journalists [18–20, 22]. Other concerns relate to transparency about whether AI generated information is used or not, and the lack of transparency about which texts were used for training the models [5]. It is, for example, unknown if copyrighted texts have been used to train the models [18, 19]. A story about the New York Times and OpenAI regarding copyright infringement [23] discussing consequences for journalism, illustrates these concerns and in this case, it was concluded that newspapers’ code of conduct should provide guidelines to whether using AI aligns with the principles explicated [18, 23].

Additionally, in the recent past, the COVID-19 pandemic has influenced the quality of reporting, and more generally the science-media relationship, since it caused a strong need for scientific information due to the novelty of the virus in combination with its societal impact [24]. Science journalists were important in bringing the scientific evidence into the public domain and giving meaning to the available scientific information. The pandemic led to an overload of scientific information, which was partly made available before peer review and via pre-prints, cf. [25]. For example, survey results from Masserani et al. found that about 55% of the journalists reported using pre-print materials in their reporting [26].

At the same time, increasing amounts of mis—and disinformation, not only during the pandemic, have led to growing concerns about the quality of science reporting [6]. Misinformation, incorrect information spread accidentally–and disinformation–incorrect information deliberately communicated–are increasingly spread, also via social media. During the pandemic, fake news became a prominent source of worry. Scheufele and Krause discussed implications of mis- and disinformation for individual citizens as well as for the group-level and societal level [7]. Consequently, they called for more systematic research on media environments.

## Materials and methods

Considering the developments above, we formulated the following research questions:

1. How is quality in news reporting reflected in the content of news articles about AI in Belgium, Italy, Portugal and Spain?
2. How do journalists in these countries, within the context of current developments of AI and the past developments in the field, deal with quality of science journalism in practice?
3. What can be learned from comparing the media ecosystems regarding the quality of science journalism in Belgium, Italy, Portugal and Spain?

To answer the research questions, we conducted two studies, with first of all, an analysis of news articles about AI in samples from Belgium, Italy, Portugal and Spain (n1 = 1474; n2 = 502). This analysis included an in-depth qualitative content analysis of 10 articles from each country. Particularly, the content analysis focused on how the media communicated about AI and, furthermore, by means of which indicators quality was reflected in the reporting. Second, semi-structured interviews were held with journalists (n = 16, four from each country) to gather insights on their views on and experiences with the quality of science journalism in relation to the recent developments with AI. The content analysis provided insights in the quality of news reporting about AI. Interviews allowed for follow-up questions and in-depth answers on the science journalism practice. In all, the mixed methodology allowed for better contextualised findings and conclusions [13].

### Principles for quality of science journalism as the basis for analysis

Our studies were guided by principles for quality science journalism which were co-created, identified, and validated during two series of workshops which were organised as part of the ENJOI-project [27]. Experts in science journalism, science communication as well as researchers (scientists), citizens, other producers, and users of science communication such as activists, teachers, or publishers participated in the workshops. The overarching principles and the related standards for quality science communication can be measured and monitored by indicators which serve as concrete characteristics of the principles and standards. The list of principles (Table 1) consists of integrity, rigour, relevance, sources of information, engagement, understanding one’s audience, accessibility, and impact. In our studies we either identified whether those principles were included in news articles or asked journalists’ views and practices regarding principles for quality science journalism.

**Table 1:** List of principles.

Principle with short description
1. **Integrity**: “Clear and explicit statement both on the **relationship with the sources** or in case of any **conflicts of interest**”
2. **Rigour**: “The information is based on **facts and data** with references, links, sources for each piece of data/fact”
3. **Relevance**: “The content explicitly explains **why the topic is of public interest and what is the novelty** compared to previous knowledge” or “There is **an explicit link between the story** and the piece of research (basic/local/national) behind it”
4. **Sources of information**: “Sources must be **clearly stated and accessible**, at least to the point of being findable
5. **Engagement:** “Response to the **Call to Action (**for example, to receive feedback; to fill in surveys and questionnaires; to participate in a training course or a live activity)”
6. **Understanding one’s audience**: “Data analytics and other quantitative measurements (surveys, questionnaires) that define the demographics, psychographics, geographical and cultural segmentation of the users”
7. **Accessibility**: “Inclusion of diverse perspectives and expertise in terms of voices and types of experts and non-experts included in the story”
8. **Impact**: “Use of selected target metrics or KPIs (depending on the media: number of followers, reach, interactions, likes, shares, number and quality of comments, number of visits, unique views, viewing or reading time)”

[Source: 27].

### Analysis of news articles about AI

The analysis of news articles about AI followed a stepwise approach. Via a systematic search in the database NexusUni, news articles from the period of 1 September 2022 to 28 February 2023, a period including the launch of ChatGPT, were gathered in CSV format in excel, and data was cleaned and restructured both semi-automatically and manually to ensure the data of interest was correct, resulting in a sample of 1474 news articles in total. Nexus Uni is a database that provides access to thousands of news, legal and business sources. It covers local, regional, national and international newspapers including publications in Belgium French, Italian, Portuguese and Spanish. Search terms and selection criteria are available in Table 2.

**Table 2:** Search terms and selection criteria.

**Search terms for all four languages together as well as in English.** Motivation to include English: for reporting purposes but also relevant since the use of English terms is common in the fields of sciences and AI. Science OR Research OR Academia OR Study OR Professor OR Researcher OR Ciencia OR Investigación OR Academia OR Estudio OR Profesor OR Docente OR Investigador OR Científico OR Scienza OR Ricerca OR Accademia OR Studio OR Science OR Recherche OR Académie OR Étude OR Ciência OR Investigação OR Academia OR Estudo OR Professor OR Professora OR Investigador OR Investigadora **AND** “Artificial intelligence” OR “Machine learning” OR “Expert system” OR “Neural network” OR “Inteligencia artificial” OR “Aprendizaje automatizado” OR “Sistema de expertos” OR “Red neuronal” OR “Intelligenza artificiale” OR “Machine learning” OR “Intelligence artificielle” OR “Apprentissage automatique” OR “Chat GPT” OR Midjourney OR Bert OR GPT3 OR “Language model” OR “natural language processing” OR “Modelo de lenguaje” OR “procesamiento natural del lenguaje” OR “modello linguistico” OR “Modèle de langage” OR “Embodied AI” OR “Embodied robot” OR “computer vision” OR “facial recognition” OR deepfake OR “Inteligencia artificial integrada” OR “visión por computación” OR “reconocimiento facial” OR “Intelligenza artificiale incorporata"” OR “visione computerizzata” OR “riconoscimento facciale” OR “Intelligence artificielle intégrée” OR “vision par ordinateur” OR “reconnaissance de visage” OR “Artificial intelligence” OR “Aprendizado de máquina” OR “aprendizagem automática” OR “Sistema inteligente” OR “Rede neura” OR “Modelo de linguagem”
**Searches were conducted within the database NexisUni within the category of NEWS** **Results were narrowed down by:** • **Languages:** French, Italian, Portuguese or Spanish. For each language separate searches were conducted. Motivation: these are the four languages included in the studies. • **Time frame:** Between 1 September 2022 and 28 February 2023. Motivation: In a half year period it may be possible to show developments regarding AI and see that reported in the publications. The period included the announcement of news about Generative AI such as ChatGPT. • **Newspapers or Blogs or Magazines & Journals:** Only results were selected that came from newspapers, blogs, magazines or journals. • Motivation: In the study we were interested in the views that journalists have on journalism and how they bring that into practice, therefore, information sources such as press releases which are produced by companies and not by journalists can be excluded. • **Geographical area:** Results were limited to the geographical area of the study in various steps. Results were limited to Europe; thereupon, to the European Union Member States and then to the countries Spain, Portugal and Italy. For the French speaking part of Belgium, the selection was narrowed down via the area Benelux, and the country Belgium. Motivation: only the geographical areas studied were included because the perspectives from these countries are underrepresented in the literature about the science-media relationship. • **Exclude Stock Stories or Exclude Obituaries:** Stock stories or obituaries were excluded. Motivation: these type of results are not relevant for the study. • **Group duplicates:** The option to exclude group duplicates was included (On). Motivation: automatic detection of group duplicates provides a cleaner sample.

**Fig 1:**
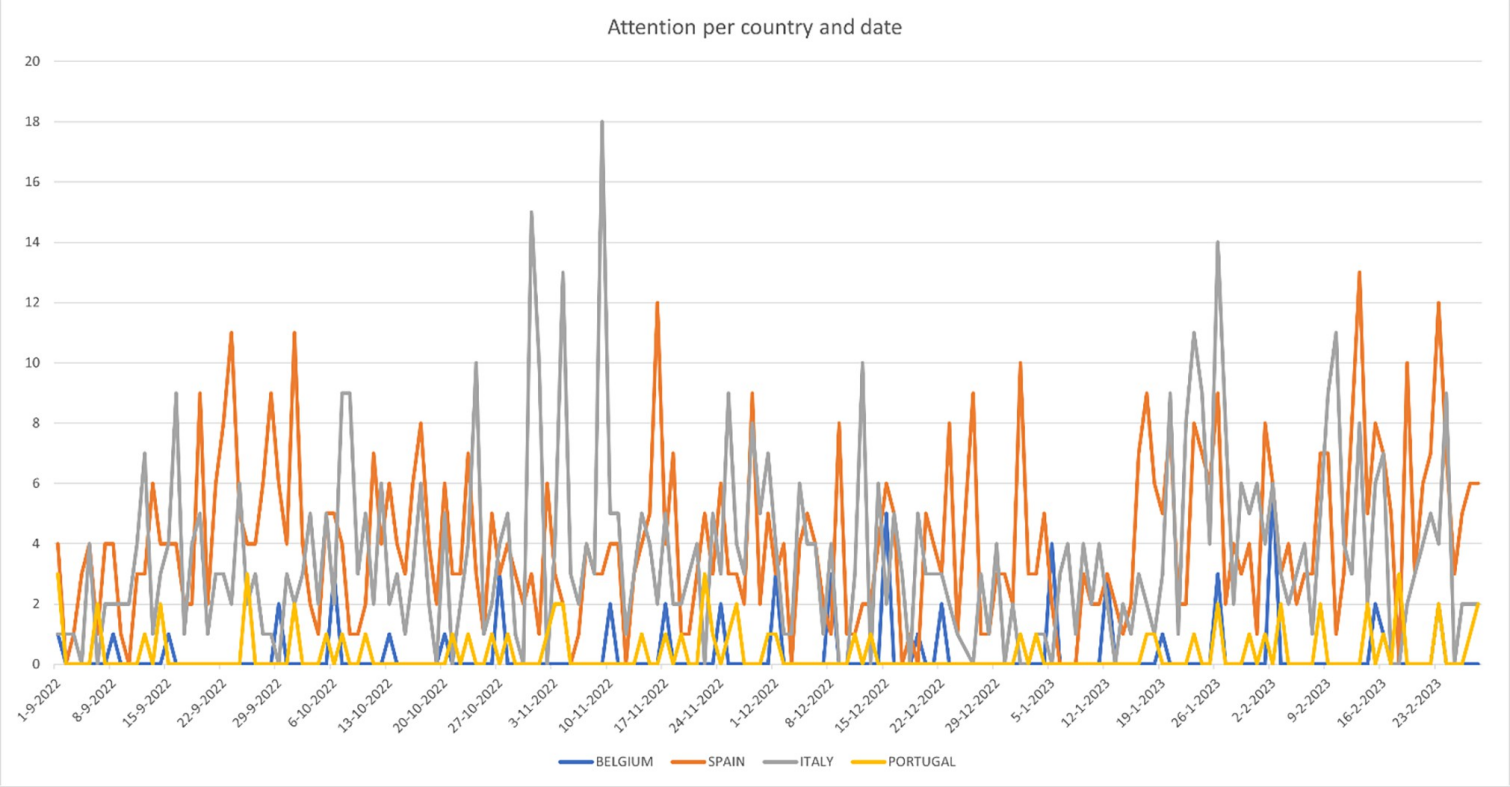
Attention to AI and science per week for Belgium, Spain, Italy and Portugal in number of articles based on the first selection sample. Selection period runs from 1 September 2022 till 28 February 2023.

For each country, peak moments of media attention were defined per week (See results section, Fig 1). Manually, based on a full text download, a second round of cleaning was performed by coders mastering the language, and duplicates or identical articles published in different editions of news outlets were removed, as were articles from sources from other countries, articles that after close reading did not include science reporting about AI or reported only about applications, economic topics, or people working in AI. This led to a total sample of 502 articles. To get an indication of the variety of the reporting about AI as well as the importance, number and type of sources; average word length and page numbers were identified.

For the content analysis, an excel template was developed by the team to enable a qualitative *systematic and comparable* in-depth analysis of ten news articles per country. Coders, team members with a background in science journalism and mastering French in Belgium, Italian, Portuguese, or Spanish, conducted the analysis, whereafter results were discussed within the team until agreement about the results was reached. A full overview of the topics included in the in-depth analysis and the operationalisation into questions and answer categories is available in Table 3. Topics were derived from literature and discussions within the team.

**Table 3:** Template for the in-depth content analysis of the newspaper articles.

	Question	Answer categories
*Reporting about AI*		
Effects of AI on society	Is there a mention of EFFECTS of AI on society?	Y/N
Effects of AI on (science) journalism	Is there a mention of EFFECTS of AI on the field of (science) journalism?	Y/N
*General characteristics*		
Sentiment	What is the sentiment in the article?	From very positive to very negative on a five-point scale
Tone of voice	What is the tone of voice?	From very optimistic to very pessimistic
Balance: different perspectives on topic	Is the topic presented from different perspectives?	From not at all to very much
Balance: interests of stakeholders reported	Are interests of different stakeholders reported?	From not at all to very much
*Explicit reference to quality*		
Mention quality science journalism and how	Is there a mention of quality of (science) journalism in general?	Y/N
Mention journalistic norms or values and how	Is there a mention of journalistic norms or values?	Y/N
Explicit reference to principles, standards or indicators and how	Is there an explicit reference to principles, standards or indicators?	Y/N
*Inclusion of principles*		
Rigour: references, links and sources	Are there references, links, sources in the article?	Y/N
Sources of information: number	What is the number of sources that are mentioned?	None, one, two, more than two
Type of sources	What type of sources are mentioned in the article?	None are mentioned, researchers from a university, researchers from industry, experts in general, studies (literature), governmental sources, developers of AI, citizen sources (lay person experiences), other authority, other (describe), multiple (describe)
Findable sources	Are sources findable? Such as a link or name of researcher or institute	Y/N/some of them
Accessibility: including voices experts and non-experts	Are voices of experts included in the story? Are voices of non-experts included in the story?	Y/N
Accessibility: addressing researchers	In what way are researchers mentioned in the article?	Not applicable; as expert in the field; as other type of expert; as educator or promotor of AI; as autonomous scientist; as critical voice of AI; as policy adviser; as public communicator
Accessibility: addressing journalists	In what way are journalists mentioned in the article?	Not applicable; as critical gatekeeper or watchdog; as civic educator; as science populariser or cheerleader; as independent reporter; as trustworthy source; as agenda setter; as public intellectual
Understanding one’s audience	Are the users (audience) identified with data in the article?	Y/N
Relevance: interest of topic; novelty; relation to research	Does content explicitly explain why the topic is of public interest? Does content explicitly explain what the novelty is compared to previous knowledge? Is there an explicit link between the story and the piece of research?	Y/N
Integrity: interest declared	Is there a statement of a potential conflict of interest?	Y/N
Engagement: call to action	Does the piece include a call to action? For example, to receive feedback; to fill in surveys and questionnaires; to participate in a training course or a live activity.	Y/N
Impact: followers, likes, comments, shares	What are the number of followers? What are the number of likes? What are the number of comments? What are the number of shares?	Fill in numbers

First, two questions identified whether reporting in the news articles referred to effects of AI on society or on journalism generally. We included these questions to identify whether (science) journalism would be a topic of discussion in the news output in comparison to reporting of societal effects.

Second, general characteristics were collected get an idea of the news sentiment in the article, that is to get a general idea of the views and feelings on the topic [28]. Sentiment analysis, mostly used as a computational linguistics technique, can distract the main opinions in a large amount of data [28]. In news output, its sentiment can influence the way readers process information and therefore, it can be used to understand how a public perceives a topic, or what public attitudes are [28]. In our qualitative analysis we included four questions which together aimed to address news sentiment. Coders scored sentiment in the whole article on a five-point scale from very positive to very negative. Similarly, the tone of voice was assessed from very optimistic to very pessimistic. Finally, balance was assessed by two questions. Balance according to Clarke [29] relates to giving attention to all sides of an issue and impartially presenting facts. In practice, journalists often don’t have time nor expertise to identify all viewpoints and therefore they emphasize the two most influential perspectives which are presented as opposites [29]. The mechanism giving equal space to voices in favour and against a point of view, can explain unbalanced information [29, 30]. However, tackling false balance is challenging, amongst others, because of the changing media landscapes. Balance was operationalised by inquiring whether the content was presented from different perspectives, and whether interests of stakeholders were reported. Both were scored from not at all to very much.

Third, explicit references to quality were included by questions about references to quality of (science) journalism in general; journalistic norms or values; and references to principles, standards or indicators. Answers could be yes or no. We added the questions since implications for journalism were more explicitly discussed in news outputs since the introduction of ChatGPT in other countries.

Finally, we operationalised assessment of the inclusion of principles, based on indicators derived from the co-creation process in the project [27] and a literature review [11]. *Rigour* was operationalised by a question if references, links or sources were included in the article. The *inclusion of sources* and how these were included was operationalised, first, by the number of sources that were mentioned (none, one, two or more than two); by the type of sources (none are mentioned; researchers from a university; researchers from industry; experts in general; studies that is literature; governmental sources; developers of AI; citizen sources; other or multiple sources) [11], and finally, if the sources were findable since a link or similar was added (yes, no or some). The principle of *accessibility* was operationalised by questions about including voices of experts or non-experts (both by yes or no); in what roles researchers were addressed (not applicable; as expert in the field; as other type of expert; as educator or promotor of AI; as autonomous scientist; as critical voice of AI; as policy advisor; as public communicator) [11]. Similarly, roles of journalists when included in the article were assessed (not applicable; as critical gatekeeper or watchdog; as civic educator; as science populariser or cheerleader; as independent reporter; as trustworthy source; as agenda setter; as public intellectual) [11]. The principle of *understanding one’s audience* was operationalised by a question whether users (audiences) were identified with data in the article (yes; no). The *relevance* principle was operationalised by inclusion of an explanation whether the topic is of public interest; the content explicitly explained the novelty compared to the previous knowledge; and the inclusion of an explicit link between the story and the underlying research (yes; no). *Integrity* was operationalised by a question whether a statement of potential conflict of interest was given (yes; no). *Engagement* was operationalised by a reference to a type of action. Finally, *impact* was operationalised by numbers of followers; likes or comments and shares. However, the NexusUni data did not include this type of information and therefore no results for this principle could be included.

From each country, 10 news articles from various sources with various lengths and publication dates were selected by the coders and coded. Articles with fewer than 250 words were excluded from the selection since these would be too short to provide in-depth information. For each news article, basic details are listed with headline, source, page number, length in words and date of publication (Supplemental materials S1 Table).

### Interviews

Interviews were prepared systematically to follow a thematic analysis [31]. To recruit the participants for the semi-structured interviews, partners from the ENJOI-project provided contact details of journalists working in the four countries. Journalists who had published about AI before, were invited to participate in an online interview via Zoom, which were conducted between 17 April and 11 July 2023. Out of the 44 journalists that were approached, 16 agreed to be interviewed, four from each country. Ethical approval for the study was provided by the Ethics Committee of the faculty of Behavioural, Management and Social Sciences of the University of Twente (nr. 230122). All interviewees read an information sheet and provided consent.

An interview scheme was prepared through an iterative process between the researchers and tested during the first interview, which did not lead to changes. Interviewees were asked to provide details about their professional background and current work, their experience with covering and using AI and the role of editorial oversight in their work. Following this, in the context of AI developments, they were asked how they assessed the quality of their own work and if they used specific quality criteria. Finally, after a short explanation about the principles, standards and indicators as these were identified in the project, they were asked to reflect on the principles. Follow-up questions were asked to get more in-depth information and to avoid misunderstandings.

Interviews were conducted in English. Verbal transcription of the interviews took place using Amberscript software and manual correction. Thereupon, a thematic analysis was applied using Atlas.ti software and main themes and sub themes were identified and coded both deductively, derived from the questions, and inductively from the transcripts. For this purpose, a codebook was developed through an iterative process between the researchers, with additional feedback from the team. Codes were applied on paragraph level for various main topics: AI, experiences, editorial oversight, quality of science journalism, science-media relationship and principles for quality. Thereupon, we actively searched for themes and patterns across the data in relation to the developments in the media ecosystems and interpreted the reported experiences, meanings and realities provided in the interviews [31].

Interviews lasted between 23 and 63 minutes with a mean of 46 minutes. Interviewees had different levels of experience with science journalism ranging from less than 5 years to more than 30 years. Eight of the interviewees worked as a freelance science journalist, and sometimes also as a science communicator, editor, consultant, or data journalist. Eight interviewees were employed as a science journalist, editor, or technology journalist. Most of the interviewees had a background in science journalism, journalism or science communication, sometimes supplemented with a background in biology, pharmacy, or space engineering. Eight participants identified as female and eight as male.

#### The context of unique media landscapes

As described in the framework above, media landscapes are unique and influence the ability of science journalists to fulfil their main task of reporting quality science information and offering contexts. In Table 4 an overview of the media landscapes in Belgium, Italy, Portugal, and Spain is provided. For Belgium, we analysed news articles in French and described the media landscape accordingly.

**Table 4:** Overview of media landscapes for Belgium (French speaking), Italy, Portugal and Spain.

*Characteristics*	Belgium (French speaking)	Italy	Portugal	Spain
*General description*	Two separate media landscapes exist (French and Flemish); not much interaction	Dominated by television; plurality of news; active contribution to the public debate	Plural, diverse; high diversity of newspapers and magazines; dominant role for television;	Plural, diverse; variety of outlets in the official languages; political affiliations influence editorial stance and coverage
*First language by inhabitants*	40% French (4.5M inhabitants); 59% Flemish (6.5M); 1% German	Italian for majority (60M inhabitants)	Portuguese (10M inhabitants); Mirandese (small minority)	Castilian Spanish (83% of 47M inhabitants); Catalan/ Valencian (12%); Galician (3%); Basque (1%).
*Embedding*	Influence by role of state	Influenced by politics	Social networks increasingly important sources for news	Large influence of 2008 economic crisis; influencing other media markets since Spanish is second most spoken language in the world
*Media culture*	One of most liberal with press council, code of ethics	Journalists are required to become members of the Order of Journalists (Ordine del Gionalisti); after passing exam	Mainly regional or local; digital media growing; less distinction traditional and online journalists	Tendency to comment rather than inform; low number are member of journalism associations with negative influence on their working conditions
*Offer media outlets*	Mainly print media; diverse offer of newspapers but decreased by steep decline of readership; few publishing companies	Many publishers with one newspaper; national level; local newspapers with high circulation numbers; recently significant drop of circulation and incomes from advertising	High number of registered publications; numbers dropping considerably	Concentrated media ownership; significant growth of digital media; online readership; loss of circulation and readership
*Main titles*	Le Soir, Sudpresse, De Tijd/L’Echo	Corriera della Sera; Repubblica; La Stampa; Il Sole 24 Ore; Il Giornale;	Correio da Manha; Jornal de Notícias; A Bola (sport)	El País; La Vanguardia; El Mundo
*Radio / TV*	Radio is fragmented and commercial; TV mainly commercial and French channels; minor role for public service broadcaster	Television dominates in comparison with other media platforms	High number of radio stations	Television market dominated by few media groups

Source Dijkstra et al, p.12-15; p.31-32 [32].

Belgium–With three official language communities, the organisation of the political system is divided among the two main language communities as is the media landscape. These do not interact much with each other [33]. French was the original cultural language of the elite which is why French also dominated the print media. Belgium has one of the most liberal press regimes, characterised by a diverse offer of newspapers reflecting ideological diversity. A press council and codes of ethics are effective although editorial statutes in print media are rare. Since the 1960s, circulation of newspapers and readership has seen a steep decrease, particularly regarding the French speaking news media. As a consequence, diversity has been shrinking with the press controlled by only a few publishing companies [33].

Italy—The Italian media system is dominated by television in comparison with other media platforms. According to Mancini and Gerli [34], audiences often turn to television for mainstream reporting. Politics has always shaped the media landscape. Unique is that all Italian journalists are required to become members of the Order of Journalists (Ordine dei Gionaristi) for which they have to pass an exam [34]. Still, the media offer a plurality of news. Many publishers publish only one newspaper. Local newspapers have less influence, however, some reach circulation numbers as high as the national newspapers [34]. In recent years, the total numbers of daily circulation of newspapers have dropped significantly as well as incomes from advertising.

Portugal–The media landscape can be considered plural and diverse with a high variety of newspapers and magazines of which many operate at the regional or local level. Portugal has a limited number of national newspapers while the number of radio stations (both local or regional) are high [35]. The influence of digital media is continuously growing. Incomes from advertisement are decreasing significantly as are numbers of circulation of newspapers. Social networks are growing sources for news making the distinction between traditional and online journalists less clear [35].

Spain–A variety of outlets in various of the official languages of Spain leading to a diverse and pluralistic media landscape is characteristic in Spain. The media landscape includes traditional print and digital platforms as well as language-based and regional media. The economic crisis of 2008 led to a loss of circulation and advertisement incomes. Other characteristics are that journalism in Spain tends to rather comment than inform while few journalists are members of journalism associations which is affecting their working conditions negatively [36]. Media ownership is concentrated to a few owners while outlets often have political affiliations or leanings.

In sum, the landscapes vary in each country, leading to a diverse set of landscapes influenced amongst others by (political) history; the number of inhabitants speaking the language, or the role of institutionalised journalism in the country. In all countries, a decline in offer and readership is noted, leading to, amongst others, lack of funding and loss of advertisements, which is challenging the position of science journalism.

## Results

In this section, the findings for the analysis of the news articles and the content analysis are described, upon which the findings from the interviews are provided.

### Findings news analysis

Table 5, below, includes the basic details of the news articles that fulfilled the inclusion criteria. The total number of the articles vary per country in the first sample (between 55 to 728 with a total of n = 1474) as well as in the second sample of articles (between 12 and 278 with a total of n = 502). Therefore, direct comparisons need to be interpreted in the context of the media landscapes. Amongst others, the number of inhabitants per country and speaking the language may influence the number of available sources, the number of readers and thus the number of publications. Thus Italy and Spain with larger populations speaking the language, offer news from more sources and with more publications. The number of sources in each country varies from 3 to 21, with the most different sources being available in the Spanish sample. Average word length varies from 414 to 1,142 words, as well as range of the length within the articles.

**Table 5:** Overview basic details of news articles per country.

Country	Sample 1 (N)	Peak moments sample 1 (month.year)	Sample 2 (N)	Appearance in # of sources	Average word length (and range)	Page # available/ not available / # page 1
*Belgium*	55	11.22 / 12.22 / 02.23	12	3	1,142 (427–2,560)	12/12/0
*Italy*	628	11.22 / 12.22 / 01.23 / 02.23	182	7	414 (40–1,883)	182/182/1
*Portugal*	63	11.22 / 02.23	30	8	795 (239–3.536)	2/28/1
*Spain*	728	09.22 / 11.22 / 12.23 / 01.23 / 02.23	278	21	934 (42–3,752)	93/185/0

Regarding attention for AI, Fig 1 show the distribution of the news articles about AI per country and does not show major peak moments in the reported period (from 1 September 2022 till 28 February 2023). In Table 5, main data is presented, and the limited number of news articles that appeared on the front page does not indicate major attention for the topic of AI either. In all, it can be concluded that the launch of ChatGPT on 30 November 2022 received some attention, but not overwhelmingly. In all four countries, most attention was given to AI in November and December 2022 and again in February 2023, moments when more news about ChatGPT was launched. However, the samples differ much which does not allow for further comparisons.

### Findings content analysis

The in-depth content analysis of 10 news articles per country added more context and depth to the characteristics of news reporting about AI. Table 6 presents the results.

**Table 6:** Results in-depth analysis of country samples.

	Belgium	Italy	Portugal	Spain
*Reporting about AI*				
Effects of AI on society	10Y	9Y/1N	9Y/1N	9Y/1N
Effects of AI on (science) journalism	1Y/9N	10N	1Y/9N	1Y/9N
*General characteristics*				
Sentiment	4 mainly positive 5 positive 1 neutral	7 mainly positive 2 positive 1 mainly negative	2 mainly positive 4 positive 2 neutral 1 negative 1 mainly negative	2 mainly positive 2 positive 4 neutral 2 negative
Tone of voice	2 mainly optimistic 5 optimistic 1 neutral 1 mainly pessimistic	7 mainly optimistic 2 optimistic 1 mainly pessimistic	2 mainly optimistic 4 optimistic 3 neutral 1 mainly pessimistic	2 mainly optimistic 3 optimistic 3 neutral 2 pessimistic
Balance: different perspectives	2 not at all 5 to some extent 3 to a considerable extent	7 not at al 3 to some extent	5 not at all 4 to some extent 1 to a considerable extent	2 not at all 5 to some extent 2 to a considerable extent 1 much
Balance: interests of stakeholders reported	7 not at all 3 to some extent	1 not at all 7 to some extent 2 to a considerable extent	4 not at all 5 to some extent 1 to a considerable extent	5 not at all 3 to some extent 1 to a considerable extent 1 very much
*Explicit references to quality*				
Mention quality science journalism	10N	10N	1Y/9N	1Y/9N
Mention journalistic norms or values	10N	10N	1Y/9N	2Y/8N
Explicit reference to principles, standards or indicators	10N	1Y/9N	1Y/9N	10 N
*Inclusion of principles*				
Rigour	8Y/2N	9Y/1N	7Y/3N	9Y/1N
Sources of information	1 none 5 one 2 two 1 more than 2	1 none 6 one 2 two 1 more than 2	3 none 3 one 3 two 1 more than 2	1 one 2 two 7 more than 2
Type of sources	2 none are mentioned 4 researchers from a university or institute 1 researchers from industry 2 studies (literature) 1 experts in general	1 none are mentioned 4 researchers from a university or institute 1 researchers from industry 2 experts in general 1 developers of AI 1 other authority	3 none are mentioned 3 researchers from a university or institute 3 researchers from industry 1 studies (literature)	2 researchers from a university or institute 2 researchers from industry 3 experts in general 1 developers of AI 1 governmental sources
Findable sources	4Y/5 some/1N	9Y/1N	5Y/1 some/4N	10Y
Accessibility: voices of experts and non-experts	Experts : 9Y/1N Non-experts : 10N	Experts : 9Y / 1N Non-experts : 2Y / 8N	Experts : 7Y/3N Non-experts : 10N	Experts : 10Y Non-experts : 1Y/9N
Accessibility: addressing researchers	4 not applicable 5 as expert in the field 1 as critical voice	1 not applicable 9 as expert in the field	3 not applicable 5 as expert in the field 2 as educator or promotor of science	2 not applicable 6 as expert in the field 2 as critical voice
Accessibility: addressing journalists	10 not applicable	10 not applicable	9 not applicable 1 as agenda setter	7 not applicable 1 as public intellectual 1 as independent reporter 1 as trustworthy source
Understanding one’s audience	10N	10N	10N	1Y/10N
Relevance: addressing interest of topic; novelty; relation to research	Topic: 10Y Novelty: 5Y/5N Research link: 3Y/7N	Topic: 9Y/1N Novelty: 9Y/1N Research link: 5Y/5N	Topic: 8Y/2N Novelty: 7Y/3N Research link: 3Y/7N	Topic: 8Y/2N Novelty: 9Y/1N Research link: 2Y/8N
Integrity: interest declared	4Y/6N	10N	2Y/8N	10N
Engagement: call to action	2Y/8N	2Y/8N	10N	1Y/9N
Impact: followers, likes, comments, shares	NA	NA	NA	NA

N = No; Y = Yes; NA = Not Available / Not applicable

#### Reporting about AI

Effects of AI on society was addressed in almost all news articles (37 out of 40), while effects on (science) journalism were rarely included (3 out of 40). Effects of AI on society were reported in terms of help or use, impact or pros and cons, change, research or education, controversy, or responsibility. The three articles including effects on (science) journalism reported these in neutral terms as one of the many professions that are affected by AI, as pros and cons of ChatGPT and as the use of AI for journalistic research or information dissemination.

#### General characteristics

Sentiment was mainly positive or neutral in all articles (34 out of 40) while only one or two articles per country were assessed as negative in sentiment. The same applies for assessment of the tone of voice. In total, 5 out of 40 articles were assessed as pessimistic or mainly pessimistic, while the remainder had a neutral, optimistic, or mainly optimistic tone of voice. Balance indicated by including different perspectives on the topic, showed that the articles were considered to report in an inclusive way ‘to some extent’ in about half of the articles. The Italian sample was considered the least balanced. Second, balance indicated by reporting interests of stakeholders, showed a few differences between the samples. In the Belgian sample, this balance was considered least included while the interests of stakeholders were included in the majority of the Italian sample and in half of the cases in the Portuguese and Spanish samples.

#### Quality of (science) journalism

Rarely in the news articles an explicit reference to quality of reporting was made (3 out of 40) which included lack of references to journalistic norms or values (3/40) or explicit references to any principles, standards, or indicators (2/40).

#### Inclusion of principles

*Rigour* was addressed by adding references to links or sources, which was found in almost all articles with exception of a few articles from the Portugues sample (33/40). The number of *sources* that was referred to was one or more in the majority of the samples (34/40).

Detailing the *type of sources*, a reference was made to studies as a source, or a general reference was made to experts, developers of AI, or governmental sources. Additionally, researchers were mainly addressed as experts in the field, or not brought up at all (10/40). In five articles, out of the 40, they were assigned a role as a critical voice, or educator, or promotor of science. Journalists, when addressed (4/40), had a role as agenda setter, public intellectual, independent report, or trustworthy source.

For the majority of the sources a *findable* reference was added (28/40).

*Accessibility* by including voices of experts and non-experts showed that articles mainly included voices from experts (35/40) and hardly from non-experts (3/40). Voices of journalists were included in 4 out of 40 articles as agenda setter, public intellectual, independent reporter and trustworthy source.

Regarding identifying audiences in the news articles as part of *understanding one’s audience*, such a reference to the audience was found in only one article (1/40).

*Relevance*, identified as interest of the topic, was added in almost all articles (35/40), novelty in the majority of the articles (32/40), while a relation to research was found in 13 out of the 40 articles.

A declared *interest* was available in 6 out of 40 articles.

*Engagement*, identified as a call to action, was found in 5 out of 40 articles.

While finally, *impact* of the articles, as number of followers, likes, comments or shares, was not identifiable since these data were lacking from the NexisUni data.

### Findings from the interviews

In this section, the interview findings are presented based on the thematic analysis which led to three main themes concerning reflections on the media landscapes in the four countries, the impact of AI and the quality of science journalism in practice. Quotes from interviewees are added to exemplify the argument. Interviewees are identified by country (BE = Belgium; IT = Italy; PT = Portugal and SP = Spain) and number (1–4).

#### Reflections on media landscapes in current times

Media landscapes provide the context for the quality of science news and science reporting about AI. Reflecting on the media landscapes, subthemes concerned the status of science journalism in each country, experiences of the interviewees and challenges they encountered. In Belgium, professional science journalism is considered challenging due to low availability of science publications. One participant (BE1) explained that only two online magazines are published (one in Dutch and one in French). Two newspapers in Dutch have dedicated science journalism staff while the other Dutch-language newspapers mostly publish science news based on press releases and translations from other media. The existence of more than two official languages makes it harder to reach readers, as brought in by one participant who reflected that the language barriers are excluding large parts of the population. In Italy, very few professional science journalists are found, according to the interviewees. Despite that, one interviewee (IT1) said that those few are able to provide high-quality content, especially producing for specialised media. Several Italian interviewees thought that main-stream media in Italy were lacking behind regarding science journalism as well as data journalism, as explained by one interviewee (IT3/06:15): “Worldwide, [data journalism] is growing. It’s now part of the daily routine in newsrooms. I would say in Italy we are, like, 10 years behind”. Interviewees from Portugal described the situation for science journalism as challenging as well. Only one mainstream media organisation has a permanent science editorial team with multiple full-time science journalists working on the production of science news. To cover science news, other newspapers mainly work with freelancers or generalist journalists. In Spain, the situation was considered comparable, with very few newspapers employing specialized science journalists and having specialised science editorial teams. According to one interviewee, this situation leads to a majority of science news being too positive and not critical enough (SP1).

General trends in the media landscapes, identified by the interviewees, are a *lack of funding* and *increased time pressure* for journalism and science journalism in particular. One reason for this situation is that audiences are unwilling to pay for news with the amount of free online information available. The rise of online content has deteriorated the position of the printed press in all countries, according to the interviewees. Consequently, one interviewee (SP4/24:12) said: “I would not encourage anyone to study journalism right now, because it’s very difficult to make a living. And I think that’s a big challenge and a big problem for the future.” An increasing amount of online content also influences the number of deadlines and the selection of content for science journalists. While discussing the need for a fast pace and high number of page views, which may encourage science editors to select science news based on press releases and hot topics, one interviewee (PT3/19:13) said: “But, we always need to bear in mind that when you pick something over other topics, you are making a decision and this decision has consequences for the diversity of content the readership will get in the end.” Several interviewees saw new developments in AI and generated content as a next step in ongoing developments related to the digital transition in journalism.

#### Impact of artificial intelligence

We identified three subthemes related to the impact of artificial intelligence: coverage of AI, use of AI and expectations of AI. Most of the interviewees covered AI in their products, sometimes in articles focusing purely on developments in AI and its societal impact and sometimes as part of other topics. For example, one participant (SP/01:56) wrote several articles about the use of AI in healthcare: "I’ve written several articles about how artificial intelligence is changing the landscape of the diagnosis of diseases. And there are several medical specialists, like oncologists, that are starting to use artificial intelligence." Some of the participants who had not covered AI explained that they considered it to be outside of their area of expertise or that they planned to write about AI but wanted to learn more about it first.

About half of the interviewees tried to use ChatGPT for fun, or when preparing a piece about ChatGPT. Some interviewees mentioned they had used ChatGPT for brief and simple descriptions of topics in a comparable role to Wikipedia, or for help with translation, rephrasing or overcoming a writers’ block. Other interviewees brought in that ChatGPT did not work well for their purposes. For example, it could not provide good references or sources of information and did not work well in certain languages (for example, in Arabic). Several interviewees mentioned using other AI applications, mainly for simple and repetitive tasks, such as translating and transcribing text.

Regarding the future impact expected from AI and ChatGPT, most interviewees thought that AI will impact (science) journalism but will not take over their jobs completely. One participant (SP3/13:20) explained it as follows: “Of course, ChatGPT can help you, but it can’t do your job. It’s like the internet or like other tools you can use. If you use them in a good way, they’re good and if you use them in a bad way, they’re bad.” Positive points the interviewees identified, included that AI could take over repetitive tasks, like transcription of interviews, and help with visual designs. Such tools were expected to become more commonplace in newsrooms. Negative points included concern for ethical implications, lack of transparency or black box issues and the risk of increasing misinformation and fake news. One participant (IT3) summed up a few examples: “Generally speaking, AI possesses privacy issues, bias issues, I think misuse of AI might be an issue. We’ve seen AI generated images, I’ve seen one earlier this morning, it was the Pentagon in the US burning. That’s obviously fake, but it can be used to spread disinformation.” These types of risks stimulate regulations for the use of AI in journalism. One Belgium interviewee (BE4/20:52) reflected: “[The Flemish council of journalistic deontology] already wrote a directive with two points: it can be interesting for journalists, but they have to be transparent. But the French speaking council is late and has no rule”.

#### Quality of science journalism in practice

In relation to AI and the quality of science journalism in practice, subthemes of editorial oversight, best practices and the use of principles were identified. Regarding editorial oversight in their daily work, interviewees who worked in newsrooms mentioned having discussions with colleagues and editors about the quality of their work. Several organisations also have a vision or mission in place, which usually consists of broad aims providing high quality, critical and independent (science) journalism. Freelancers reported less frequent interactions with editors but also said they sometimes received feedback from editors on their writing quality. Freelancers found other ways to ensure quality, for example by visiting conferences, discussing with colleagues or asking ‘others’ for feedback, for example, one participant (SP3/16:29) stated: “In specific areas, with more science issues, I try to have the help of an expert who can read it, I have some friends or colleagues who can read a manuscript for bigger things”.

When asked, according to the interviewees, the quality of their work is defined by: reliability and independence of sources; fact-checking; checking the readability and understandability of the writing; creativity and storytelling; speaking to multiple audiences and showing multiple points of views. Journalism ethics were considered key to science journalism, because of the large impact it can have, for example on decisions that people make about their health. Quick deadlines, however, may hinder taking the extra steps for guaranteeing quality, as said (IT2/4:14): “Another thing I do to double-check my work on some occasions, I send it to two or three people who are competent in the field. But this doesn’t happen often because I don’t have time”. AI applications as ChatGPT could help journalists to write understandably without simplifying scientific information too much. A few interviewees mentioned they had already used ChatGPT to help them rephrase sentences and find synonyms for complex or overused words.

Best practices in the journalistic methodology related to fact-checking, being aware of biases (including false balance), sharing data, sticking to facts and taking sufficient time. Aligned with reported behaviour for ensuring quality of reporting, interviewees described contacts with informed and independent sources as best practice. Transparency towards sources is leading in these contacts. Several journalists worried that this will become more complicated as AI will be used more, as one interviewee explained: "If you ask AI something you don’t know what the source is, so it can be even more complicated as well" (IT1/39:38). Another best practice was to pay attention to scientific consensus, which, for some, meant that journalists should have some basic scientific knowledge when reporting about topics. In particular, some interviewees mentioned contrasting information and adding different points of view on every topic as best practices, whereas others emphasized the need to avoid *false balance*: “In science journalism, I think one of the most important is not to believe the minority. In political journalism it is normal to put one pro and one contra in the discussion, but it doesn’t work in science journalism. If you have 99 scientists who say that it is like this, and you have one scientist who doubts it. Then it is not a good idea to represent these 99 scientists on the same level as this one exception” (BE1/41:19).

Reflecting on the principles which guided the discussions about quality, interviewees thought that integrity, rigour, sources, understanding one’s audiences and relevance were the most important ones. They found these principles closely related, especially integrity and rigour, but one interviewee added sources as well (PT2/20:05): “For the news in general, I think it always has to have rigour. And then if it has rigour, it has to have good sources of information. [The third one] would be integrity. (…) I’d say those three go more or less in the same line”. Relevance, selecting as a journalist what is important for the reader, was considered another main principle. When pressed, the interviewees mentioned engagement, impact, and accessibility as less relevant principles, with some seeing this as a responsibility of editors instead. In a role of editor, interviewees tended to emphasise the importance of relevance and timeliness of news stories more than other interviewees. When asked, the interviewees suggested adding principles as fairness, independence, diversity, equity and inclusion, creativity, empathy, and transparency, in the meaning of being honest about mistakes.

Finally, regarding potential impact of AI on the principles for high-quality science journalism, most interviewees agreed that the principles suffice also for AI (SP1/42:08): “Maybe some stuff will be more difficult. But I don’t think it will change. Journalists will always have to be like this. (…) This is always going to be important”. Several interviewees agreed that developments in AI may make adherence to some of the principles more difficult. AI can make fact checking and finding reliable sources more complicated, influencing principles of rigour, integrity, relevance, and sources of information. However, positive impacts were also expected, for example by improving accessibility and engagement, and providing better data on impact and the audience.

## Discussion

Our aims were to analyse the quality of news reporting about AI (RQ1) and to study the perspective of science journalists on this topic in light of the recent developments of AI generated content (RQ2). We compared the media ecosystems for the countries Belgium, Italy, Portugal, and Spain as understudied areas in the science-media relationship (RQ3). In sum, the analysis of the news output with the in-depth content analysis provides insights in reporting styles in different media landscapes, while the interviews add insights about the changing roles for science journalism and science journalists. Developments regarding generative AI are expected to further influence the media landscape, the profession, and the quality of science journalism.

The findings show four different media landscapes and, at the same time, reporting that adheres to the same quality criteria. Continuous attention, albeit not abundantly, was given to AI during the period selected for analysis, with more news articles in countries with higher number of inhabitants than in those with lower numbers (Italy, Spain versus Belgium, Portugal). Despite the varying number of publications, in-depth analysis of the–not representative–samples of news articles found similar reporting styles in the four countries. Sentiment in the news articles was mainly positive and optimistic in tone. Different perspectives and interests of various stakeholders were often reported. This can be interpreted to be in line with recent findings from an analysis of headlines about ChatGPT in the UK showing a leaning towards explaining the technology [12].

Mainly experts as researchers from universities were included while non-experts hardly were given a voice. Our findings align with literature bringing in that science journalism generally complies with the criteria for journalism, in contrast to those of the scientific system, as stated [3]. Furthermore, past studies show that reporting about science, for topics like biotechnology or nanotechnology, is often optimistic in tone and accurate despite scientists sometimes expecting a more critical tone and sentiment, cf. [37]. Findings from a study into the use of anonymous sources, related to the case of COVID-19 news, reported a shift towards a more neutral sentiment and use of anonymous sources [38]. However, our qualitative analysis of AI news found mainly a positive sentiment and optimistic tone of voice with a balanced use of sources. An explanation could be that during the pandemic also general journalists reported about COVID-19 who may be trained to be more critical while reporting about AI in our period of analysis was still mainly the domain for science journalists.

Effects of AI on society were almost always included by researchers presented as experts in the fields. However, effects of AI on journalism–during our selected time period–were not brought up, implying a lack of explicit reference to the quality of journalism. Meanwhile, consistently, indicators for quality were found in the news articles. Rigour was included by references to links and sources for each piece; sources of information were stated and findable; accessibility by including voices of experts were found while both the relevance of the topic as well as the novelty were identifiable in the samples. In sum, similar and comparable criteria for news quality were found in the analysed samples in the four countries. Thus, in all, despite a country’s deploring media landscape, quality criteria for science journalism reporting is based on values that are shared widely within the profession of journalism, which is a hopeful perspective [3].

*Interviews* added insights in practices of science journalism in the four countries and how those practices are affected by developments in AI and ongoing changes in the media landscapes. In all countries, developments in AI are expected to impact science journalism and the quality of reporting, but this process is still in the early stages. Currently, journalists use AI tools mainly for limited, simple tasks, such as translating and transcribing, as well as for paraphrasing suggestions and inspiration. In time, it is expected that AI applications can be helpful for more tasks such as improving accessibility of news and sources, designing images, and providing better data to increase the journalists’ understanding of audiences and improve impact of news articles. Overall, journalists expressed a mildly positive view on AI, which matches the predominantly positive sentiment and optimistic tone identified in our media analysis and aligns with the optimistic motivations outlined by [11].

Nevertheless, concerns about the state of science journalism were prominent. Funding for specialised science journalism is often lacking. This lack of resources implies that journalists have a restricted amount of time available to produce science journalism content. Not surprisingly, time constraints were reported as the main hurdle to overcome in interactions with scientists, echoing findings from other studies [21, 38, 39].

Moreover, concerns exist that generative language models, as ChatGPT, may impact the profession in a negative way. Funding for specialized science journalism in newsrooms may further be reduced. Recently, a German tabloid announced a reorganisation with an expected 200 job-loss, partly due to replacement by AI, showing the pressing concerns [20]. Also, AI may negatively impact science journalism through a lack of transparency, risks of misinformation and negative ethical implications. Schäfer identified such concerns of ChatGPT in a recent discussion of influences for the ecosystem of science communication [5]. Similar concerns were also found in newspaper articles and discussions in various countries about consequences of ChatGPT for the profession of journalism [18, 19, 23].

Guidelines for principles, standards, and indicators, and discussing these within the profession, may be ways to safeguard the quality of science journalism in this new age of AI. While employed journalists discussed quality of reporting as part of their team meetings, freelancers ensured quality of reporting in other ways, by discussing with friends and colleagues or sometimes in the newsrooms or at conferences. Generally, principles are perceived as important for quality of reporting, with rigour, integrity, sources of information and understanding one’s audience as main guidelines. In contrast, engagement, accessibility, and impact were found to be less relevant in daily work practices. Interestingly, current developments in AI were thought to make it more difficult to adhere to the most valued principles of rigour, integrity and sources of information, but improve accessibility and impact.

### Limitations

One of the limitations of our study is its scope to analyse the perspective of science journalists, thus disregarding the perspective of scientists. However, the views of scientists have frequently been studied while studies into perspectives from journalists lack behind [11, 38]. Journalists, nevertheless, are important actors in the news cycle concerning science and technology. The content analysis allowed for more in-depth analysis while the mixed method approach could contextualise our findings about the quality of science journalism. In addition, the perspective of Southern European countries as well as Belgium provided relevant insights in these understudied areas in comparison to the predominantly Western (European) perspectives. Another limitation is that the interviews with the journalists from these countries were conducted in English instead of their local languages, which sometimes led to language barriers. Furthermore, the time frame of the media analysis, which was during the launch of ChatGPT, appears to have been before reflection by media producers on the AI developments increased and became prominent. In our view, it is important to get a broader understanding of the science-media relationship since developments in AI operate at a worldwide level. Future studies into understudied areas (non-Western but also including broader European perspectives as well as comparisons between countries) can add relevant perspectives.

## Conclusions

To conclude, taking the Southern European media perspective on AI during the period that ChatGPT was launched, provides valuable insights in the quality of news reporting in a changing context. The findings from Belgium, Italy, Portugal, and Spain show that, in different media ecosystems, despite challenging conditions for the science journalism profession and the changes brought upon by AI developments, the actors take their responsibility seriously by adhering to the basic quality criteria for reporting. AI influence is still in its infancy. Developing guidelines for quality journalism and discussing these within the profession may contribute to a stronger profession in the longer term. Newer forms of science journalism, as investigative journalism, engagement journalism or solution journalism may further contribute to tackling current challenges as was further explored in the ENJOI-project [39, 40]. In a context of mis- and disinformation, and the spreading of fake news, insight into quality of reporting and the journalists role is crucial, which is why more studies into this perspective will be valuable. Our findings contribute to these insights. Additionally, comparisons of science journalists and general journalists regarding reporting about science and towards AI particularly may add further insights. Finally, it may be interesting to study how journalists see their responsibilities in the relationship with researchers during the process of increasing medialisation of science.

## Supporting information

S1 Table Samples news articles. (DOCX)
