# Supplementary material for: Quality of science journalism in the age of Artificial Intelligence explored with a mixed methodology
Source: PLoS One. 2024 Jun 18;19(6):e0303367. doi: 10.1371/journal.pone.0303367 (PMC11185480; doi:10.1371/journal.pone.0303367)
Supplement: S1 Table — (DOCX) [file pone.0303367.s001.docx]

**S1 Table**

| **Samples news articles.** Overview of basic characteristics of the selected articles per country. For each country 10 news articles were selected for a manual analysis. | | | | | |
| --- | --- | --- | --- | --- | --- |
| **Country** | **HEADLINE** | **SOURCE** | **PAGE NUMBER** | **WORDS** | **DATE** |
| Belgium | [«J'ai envie d'avoir un gros impact dans l'univers des déchets» Victor Dewulf](https://advance.lexis.com/api/document?collection=news&id=urn:contentItem:66KT-7BK1-JCDM-12K5-00000-00&context=1516831&sourcegroupingtype=G) | Le Vif/L'Express | 88 | 1610 | 13-10-2022 |
| Belgium | [Les mots pour le dire Communication](https://advance.lexis.com/api/document?collection=news&id=urn:contentItem:66WB-DGT1-JCDM-126M-00000-00&context=1516831&sourcegroupingtype=G) | Le Vif/L'Express | 98 | 884 | 17-11-2022 |
| Belgium | [Dans les coulisses de l'hôpital du futur ARCHITECTURE HOSPITALIÈRE](https://advance.lexis.com/api/document?collection=news&id=urn:contentItem:670B-0YN1-JCDM-1217-00000-00&context=1516831&sourcegroupingtype=G) | Journal du Médecin (French) | 22 | 1078 | 1-12-2022 |
| Belgium | [Des "data centers" en route vers l'écologie NUMÉRIQUE](https://advance.lexis.com/api/document?collection=news&id=urn:contentItem:671T-T6X1-JCDM-12SP-00000-00&context=1516831&sourcegroupingtype=G) | Trends/Tendances | 62 | 523 | 8-12-2022 |
| Belgium | [«ChatGPT est un levier pour atteindre des résultats d'un plus haut niveau»](https://advance.lexis.com/api/document?collection=news&id=urn:contentItem:677T-0CG1-JCDM-1564-00000-00&context=1516831&sourcegroupingtype=G) | Le Vif/L'Express | 60 | 959 | 5-1-2023 |
| Belgium | [«La photo est une illusion»](https://advance.lexis.com/api/document?collection=news&id=urn:contentItem:677T-0CG1-JCDM-155R-00000-00&context=1516831&sourcegroupingtype=G) | Le Vif/L'Express | 32 | 1127 | 5-1-2023 |
| Belgium | [Donner sa langue à ChatGPT](https://advance.lexis.com/api/document?collection=news&id=urn:contentItem:6798-SP91-JCDM-10HJ-00000-00&context=1516831&sourcegroupingtype=G) | Le Vif/L'Express | 3 | 698 | 12-1-2023 |
| Belgium | [«On ne peut plus accepter que le numérique soit un no man's land» Antoinette Rouvroy](https://advance.lexis.com/api/document?collection=news&id=urn:contentItem:67D8-C6P1-JCDM-12JB-00000-00&context=1516831&sourcegroupingtype=G) | Le Vif/L'Express | 40 | 2560 | 26-1-2023 |
| Belgium | [L'IA aide à trouver des effets secondaires non connus](https://advance.lexis.com/api/document?collection=news&id=urn:contentItem:67FS-5H11-JCDM-1027-00000-00&context=1516831&sourcegroupingtype=G) | Journal du Médecin (French) | 4 | 774 | 2-2-2023 |
| Belgium | [Faut-il craindre la révolution ChatGPT? INTELLIGENCE ARTIFICIELLE](https://advance.lexis.com/api/document?collection=news&id=urn:contentItem:67FS-5H11-JCDM-107W-00000-00&context=1516831&sourcegroupingtype=G) | Trends/Tendances | 20 | 1924 | 2-2-2023 |
| Italy | [Clima, l'Ia è alleata d'impresa Uno studio su intelligenza artificiale e ambiente di AI for the Planet, Bcg e Bcg Gamma](https://advance.lexis.com/api/document?collection=news&id=urn:contentItem:669T-6HK1-DYDY-9093-00000-00&context=1516831&sourcegroupingtype=G) | ItaliaOggi7 | 18 | 953 | 5-9-2022 |
| Italy | [Retinopatia diabetica Con intelligenza (anche artificiale) oggi si può fermarla subito Dossier](https://advance.lexis.com/api/document?collection=news&id=urn:contentItem:66ND-11F1-F13X-91KS-00000-00&context=1516831&sourcegroupingtype=G) | Corriere della Sera (Italy) | 4.5 | 1380 | 20-10-2022 |
| Italy | [Ecco Leonardo, il supercomputer È il quarto più veloce del mondo Il colosso installato al Tecnopolo di Bologna ha già scalato le classifiche prima dell'inaugurazione](https://advance.lexis.com/api/document?collection=news&id=urn:contentItem:66W4-HJ91-JDN6-F4TR-00000-00&context=1516831&sourcegroupingtype=G) | Il Resto del Carlino (Italy) | 17 | 630 | 16-11-2022 |
| Italy | [Consob studia algoritmo che scopre l'insider trading](https://advance.lexis.com/api/document?collection=news&id=urn:contentItem:671D-PG51-JBYW-K04F-00000-00&context=1516831&sourcegroupingtype=G) | MF | 8 | 405 | 6-12-2022 |
| Italy | [L'intelligenza artificiale seleziona la frutta L'azienda Sorma ha ideato una nuova tecnologia destinata ad avere portata globale ottimizzando tempi e costi aziendali](https://advance.lexis.com/api/document?collection=news&id=urn:contentItem:6733-PVN1-F148-514R-00000-00&context=1516831&sourcegroupingtype=G) | Il Resto del Carlino (Italy) | 20 | 646 | 14-12-2022 |
| Italy | [Il pilota? Adesso è un robot In autodromo corre il futuro Da Indianapolis la gara di monoposto a guida autonoma](https://advance.lexis.com/api/document?collection=news&id=urn:contentItem:6781-2101-JDN6-F3BB-00000-00&context=1516831&sourcegroupingtype=G) | Il Giorno (Italy) | 2 | 476 | 6-1-2023 |
| Italy | [Prevedere le malattie con un pc: la sfida dell'intelligenza artificiale Lo studio](https://advance.lexis.com/api/document?collection=news&id=urn:contentItem:679R-NR31-F13X-913T-00000-00&context=1516831&sourcegroupingtype=G) | Corriere della Sera (Italy) | 24 | 731 | 14-1-2023 |
| Italy | [L'assistente virtuale parla con i sordi Di Iorio, ad QuestIt, presenta il prototipo dell'avatar capace di dialogare grazie al linguaggio dei segni](https://advance.lexis.com/api/document?collection=news&id=urn:contentItem:67CN-STT1-JDN6-F544-00000-00&context=1516831&sourcegroupingtype=G) | La Nazione (Italy) | 10 | 523 | 20-1-2023 |
| Italy | [Il caos del cervello? A tutte le età Uno studio italiano ha misurato i cambiamenti del ritmo delle onde cerebrali nell'invecchiamento](https://advance.lexis.com/api/document?collection=news&id=urn:contentItem:67CD-JYB1-F148-5186-00000-00&context=1516831&sourcegroupingtype=G) | La Nazione (Italy) | 13 | 581 | 22-1-2023 |
| Italy | [L'intelligenza artificiale Da Dante a Rembrandt se il computer fa l'artista All'Ai-Lab della Iulm, il laboratorio dove i robot dipingono e illustrano la Commedia](https://advance.lexis.com/api/document?collection=news&id=urn:contentItem:67D8-FRB1-JDN6-F449-00000-00&context=1516831&sourcegroupingtype=G) | Il Giorno (Italy) | 18 | 638 | 26-1-2023 |
| Portugal | [Os dados estão ficando cada vez mais verdes](https://advance.lexis.com/api/document?collection=news&id=urn:contentItem:66HM-2KJ1-DYY9-00C6-00000-00&context=1516831&sourcegroupingtype=G) | CE Noticias Financieras Portuguese |  | 1002 | 1-10-2022 |
| Portugal | [Velocidade dos passos pode indicar o estágio da doença de Parkinson](https://advance.lexis.com/api/document?collection=news&id=urn:contentItem:66P3-SK91-JDVB-H1Y4-00000-00&context=1516831&sourcegroupingtype=G) | O Estado de S.Paulo |  | 462 | 23-10-2022 |
| Portugal | [Consórcio de IA liderado por Unbabel investe 78 milhões de euros](https://advance.lexis.com/api/document?collection=news&id=urn:contentItem:66SM-8K21-JCG7-806H-00000-00&context=1516831&sourcegroupingtype=G) | CE Noticias Financieras Portuguese |  | 1060 | 3-11-2022 |
| Portugal | [Medina defende valor estratégico da Web Summit. Musiversal vence Road 2 Web Summit](https://advance.lexis.com/api/document?collection=news&id=urn:contentItem:66SV-7VR1-JCG7-80W6-00000-00&context=1516831&sourcegroupingtype=G) | Jornal de Negócios |  | 2095 | 4-11-2022 |
| Portugal | [Argentina - Portugal para ser a final, segundo a inteligência artificial](https://advance.lexis.com/api/document?collection=news&id=urn:contentItem:66W0-4751-DYY9-02FJ-00000-00&context=1516831&sourcegroupingtype=G) | CE Noticias Financieras Portuguese |  | 253 | 14-11-2022 |
| Portugal | [Arlindo Oliveira: "Quem dominar a inteligência artificial vai dominar a economia do planeta"](https://advance.lexis.com/api/document?collection=news&id=urn:contentItem:67DJ-B7J1-JCG7-83W6-00000-00&context=1516831&sourcegroupingtype=G) | CE Noticias Financieras Portuguese |  | 651 | 26-1-2023 |
| Portugal | [Investigadores do Porto criam sistema de apoio a combate de catástrofes naturais](https://advance.lexis.com/api/document?collection=news&id=urn:contentItem:67H9-NYK1-JCG7-8357-00000-00&context=1516831&sourcegroupingtype=G) | Jornal de Negócios |  | 487 | 8-2-2023 |
| Portugal | [Investigadores portugueses recebem 250 mil dólares para investigar chatbot da Amazon](https://advance.lexis.com/api/document?collection=news&id=urn:contentItem:67K8-CP21-JCG7-80TY-00000-00&context=1516831&sourcegroupingtype=G) | Semana Informatica |  | 276 | 16-2-2023 |
| Portugal | [ChatGPT: para bom entendedor, meia palavra basta?](https://advance.lexis.com/api/document?collection=news&id=urn:contentItem:67KF-VY11-DYY9-003X-00000-00&context=1516831&sourcegroupingtype=G) | CE Noticias Financieras Portuguese |  | 1622 | 18-2-2023 |
| Portugal | [Como seria Madeleine McCann aos 21 anos de idade, de acordo com a Inteligência Artificial](https://advance.lexis.com/api/document?collection=news&id=urn:contentItem:67MH-7HG1-DYY9-012G-00000-00&context=1516831&sourcegroupingtype=G) | CE Noticias Financieras Portuguese |  | 390 | 23-2-2023 |
| Spain | [Propiedad intelectual e inteligencia artificial](https://advance.lexis.com/api/document?collection=news&id=urn:contentItem:66W4-YTK1-JCN5-53K8-00000-00&context=1516831&sourcegroupingtype=G) | El Pais | 16 | 874 | 11-16-2022 |
| Spain | [Esta inteligencia artificial está metiendo miedo, pero no es para tanto: esto es lo que hay detrás](https://advance.lexis.com/api/document?collection=news&id=urn:contentItem:671F-V0N1-DXN0-71HD-00000-00&context=1516831&sourcegroupingtype=G) | El Confidencial |  | 1974 | 12-06-2022 |
| Spain | [Los focos de la ciencia en 2023: ingeniería genética, física cuántica e IA](https://advance.lexis.com/api/document?collection=news&id=urn:contentItem:676J-0601-JDRJ-N540-00000-00&context=1516831&sourcegroupingtype=G) | El Mundo | 13 | 1264 | 12-30-2022 |
| Spain | [Microsoft invertirá 10.000 millones en la empresa de inteligencia artificial OpenAI](https://advance.lexis.com/api/document?collection=news&id=urn:contentItem:67CV-YC31-F046-W4TF-00000-00&context=1516831&sourcegroupingtype=G) | El Pais | 43 | 697 | 01-24-2023 |
| Spain | [Los artífices del diseño de proteínas con inteligencia artificial ganan el Fronteras del Conocimiento Los hallazgos conseguidos por David Baker, Demis Hassabis y John Jumper permitirán desarrollar nuevos fármacos y vacunas](https://advance.lexis.com/api/document?collection=news&id=urn:contentItem:67D9-93M1-F07X-G0GC-00000-00&context=1516831&sourcegroupingtype=G) | Hoy |  | 509 | 01-26-2023 |
| Spain | [La Inteligencia Artificial afirma que el calentamiento global es inevitable](https://advance.lexis.com/api/document?collection=news&id=urn:contentItem:67GN-R9X1-DYY9-009S-00000-00&context=1516831&sourcegroupingtype=G) | Marca |  | 278 | 02-04-2023 |
| Spain | [Google vs. Microsoft : guerra abierta por la inteligencia artificial que va a cambiar tu vida](https://advance.lexis.com/api/document?collection=news&id=urn:contentItem:67HR-W8T1-JB5X-H01C-00000-00&context=1516831&sourcegroupingtype=G) | El Confidencial |  | 2965 | 02-12-2023 |
| Spain | [Desmontamos ChatGPT: muestra carencias pese a su probada eficacia](https://advance.lexis.com/api/document?collection=news&id=urn:contentItem:67JT-FYJ1-DYY9-04VV-00000-00&context=1516831&sourcegroupingtype=G) | El Economista |  | 3752 | 02-14-2023 |
| Spain | [¿Cómo pueden utilizar ChatGPT y GPT-3 las empresas?](https://advance.lexis.com/api/document?collection=news&id=urn:contentItem:67JM-K631-JBP6-P52X-00000-00&context=1516831&sourcegroupingtype=G) | CIO Magazine (Spain) |  | 2288 | 02-15-2023 |
| Spain | [ChatGPT, la 'chuleta inteligente'.Responde a cualquier pregunta,hace trabajos, resúmenes... ¿Habráque volver al boli y al papel?](https://advance.lexis.com/api/document?collection=news&id=urn:contentItem:67MG-FGC1-F07X-G4K3-00000-00&context=1516831&sourcegroupingtype=G) | Hoy |  | 792 | 02-24-2023 |
